# Prediction of pulmonary function after major lung resection using lung perfusion scintigraphy with single-photon emission computed tomography/computed tomography

**DOI:** 10.20407/fmj.2019-012

**Published:** 2020-02-11

**Authors:** Hiroshi Kawai, Toru Kawakami, Masakazu Tsujimoto, Ayami Fukushima, Satomi Isogai, Hisato Ishizawa, Hiromitsu Nagano, Takahiro Negi, Daisuke Tochii, Sachiko Tochii, Takashi Suda, Hiroshi Toyama, Yasushi Hoshikawa

**Affiliations:** 1 Department of Thoracic Surgery, Fujita Health University, School of Medicine, Toyoake, Aichi, Japan; 2 Department of Radiology, Fujita Health University Hospital, Toyoake, Aichi, Japan; 3 Respiratory Function Testing Laboratory, Fujita Health University Hospital, Toyoake, Aichi, Japan; 4 Department of Radiology, Fujita Health University, School of Medicine, Toyoake, Aichi, Japan

**Keywords:** Lung cancer, Major surgery, Predicted postoperative forced expiratory volume in 1 second (ppoFEV_1_), Predicted postoperative diffusing capacity for carbon monoxide (ppoDL_CO_), Perioperative morbidity and mortality

## Abstract

**Objective:**

Precise prediction of postoperative pulmonary function is extremely important for accurately evaluating the risk of perioperative morbidity and mortality after major surgery for lung cancer. This study aimed to compare the accuracy of a single-photon emission computed tomography/computed tomography (SPECT/CT) method that we recently developed for predicting postoperative pulmonary function versus the accuracy of both the conventional simplified calculating (SC) method and the method using planar images of lung perfusion scintigraphy.

**Methods:**

The relationship between the postoperative observed % values of the forced expiratory volume in 1 second (FEV_1_) or diffusing capacity for carbon monoxide (DL_CO_ or DL_CO_’) and the % predicted postoperative (%ppo) values of FEV_1_, DL_CO_, or DL_CO_’ calculated by the three methods were analyzed in 30 consecutive patients with lung cancer undergoing lobectomy.

**Results:**

The relationship between the postoperative observed % values and %ppo values calculated by the three methods exhibited a strong correlation (Pearson r>0.8, two-tailed *p*<0.0001). The limits of agreement between the postoperative % values and %ppo values did not differ among the three methods. The absolute values of the differences between the postoperative % values and %ppo values for FEV_1_ and DL_CO_’ were comparable among the three methods, whereas those for DL_CO_ of SPECT/CT were significantly higher than those of the planar method. Conversely, in patients with preoperative %DL_CO_’ of <80% predicted, the absolute values of the differences between the postoperative %DL_CO_’ and %ppoDL_CO_’ of SPECT/CT tended to be smaller than those of the SC and planar methods.

**Conclusion:**

The accuracy of SPECT/CT for predicting postoperative pulmonary function is comparable with that of conventional methods in most cases, other than in some patients with diffusion impairment.

## Introduction

Perioperative morbidity and mortality after major surgery for lung cancer is significantly associated with postoperative residual pulmonary function, which is determined by preoperative pulmonary function and the lung volume to be resected. Therefore, precise evaluation of preoperative pulmonary function and accurate prediction of postoperative pulmonary function are crucial for surgical patient selection.^1–6^ The predicted postoperative (ppo) forced expiratory volume in 1 second (FEV_1_) has traditionally been a fundamental parameter in the functional evaluation of surgical candidates with lung cancer. A low ppoFEV_1_ (<40% predicted) is strongly associated with increased respiratory morbidity (47%–71%) and mortality (29%–50%).^2,5,7–9^ The diffusing capacity for carbon monoxide (DL_CO_) has recently been considered important as an independent predictor of postoperative respiratory morbidity and mortality even in patients with a normal FEV_1_.^10,11^ Several studies have demonstrated that a reduced ppoDL_CO_ (<40% predicted) is associated with an increased risk of perioperative pulmonary complications (34%–67%) and death (22%–43%).^2,3,5,10,11^ Therefore, the ability to predict postoperative pulmonary function (ppoFEV_1_ and ppoDL_CO_) with increased accuracy should reduce perioperative complications and avoid postoperative deaths.

The conventional method of calculating ppo pulmonary function entails multiplying the preoperatively measured pulmonary function value with (1–number of functional lung segments to be resected/total number of functional segments), assuming the total number of segments in the bilateral lungs is 19.^7,12^ The values are then corrected for differences in pulmonary blood flow between the left and right lungs by determining the fraction of total perfusion for the resected lung based on planar lung perfusion scintigraphy images and then plugging the ratio into the equation.^13,14^ However, postoperative pulmonary function predictions with the conventional method can be inaccurate; some reports have described uneven intrapulmonary blood flow in patients with emphysematous changes or interstitial pneumonia in the background lung area.^15–17^ In recent years, several radiological methods including single-photon emission computed tomography/computed tomography (SPECT/CT), quantitative CT, perfusion magnetic resonance imaging, and an improved segmentation method using conventional planar scintigraphy images have been developed for more accurate prediction of postoperative pulmonary function even in patients with uneven intrapulmonary perfusion due to chronic lung diseases.^18–27^ However, the accuracy of these methods was mainly studied using FEV_1_. No previous studies used DL_CO_, which is an important predictive factor independent of FEV_1_.^10,11^

In the present study, we developed a method to determine the blood flow distribution to each lobe through three-dimensional analysis of lung perfusion scintigraphy images using SPECT/CT and compared this method with conventional methods in terms of accuracy in prediction of postoperative pulmonary function, including FEV_1_ and DL_CO_.

## Methods

This study included patients aged ≥16 years who underwent lobectomy (including bilobectomy but excluding pneumonectomy) for primary non-small cell lung cancer from 1 April 2018 to 1 December 2018 and pulmonary function testing approximately 2 to 4 months postoperatively at Fujita Health University Hospital. For each patient, %ppoFEV_1_, %ppoDL_CO_, and %ppoDL_CO_’ were predicted using the following three methods: simplified calculating (SC) method using Ali’s formula (SC method), planar method, and SPECT/CT. The results were compared with the postoperative %FEV_1_, %DL_CO_, and %DL_CO_’ measured 2 to 4 months postoperatively to determine which prediction method was superior. This study was conducted after receiving approval from the Institutional Review Board of Fujita Health University (Approval No. HM18-313).

### Patient characteristics

The 30 patients included in the analysis set comprised 20 men and 10 women with a mean age±standard deviation (SD) of 66±9 years (range, 37–77 years) and pathologically diagnosed non-small cell lung cancer. The final diagnosis was adenocarcinoma in 21 patients, squamous cell carcinoma in 7, adenosquamous carcinoma in 1, and pleomorphic carcinoma in 1. The surgical procedures used were right upper lobectomy in 6 patients, right middle lobectomy in 5, right lower lobectomy in 8, right upper and middle lobectomy in 1, right middle and lower lobectomy in 1, left upper lobectomy in 5, and left lower lobectomy in 4. The surgical approaches used were thoracotomy in 4 patients, 3-port video-assisted thoracic surgery (VATS) in 21, uniportal VATS in 2, and robotic VATS in 3. Of the 30 patients, 10 (33.3%) had obstructive ventilatory impairment (FEV_1_/forced vital capacity [FVC] of <70%), and 1 (3.3%) had interstitial pneumonia. Postoperative complications were observed in 5 of 30 (16.7%) patients: prolonged air leakage (defined as Clavien–Dindo classification grade ≥IIIA requiring re-drainage or pleurodesis) in 4 patients, and chronic respiratory failure requiring home oxygen therapy in 1 patient. No patient died within 30 days postoperatively. The patients’ characteristics are shown in Table 1.

### Pulmonary function testing

Preoperative and postoperative pulmonary function examinations (spirometry and measurement of single-breath DL_CO_) were performed according to the American Thoracic Society/European Respiratory Society standards^28,29^ using a total respiratory function testing system (CHESTAC-8900; CHEST M.I., Inc., Tokyo, Japan). The mean number of days between the surgery and the postoperative examination was 89±20.

### Lung perfusion scintigraphy

Each patient received an intravenous injection of ^99m^Tc-macroaggregated albumin (185 MBq) in the supine position. SPECT images and CT images were obtained sequentially, immediately following static perfusion examination with a dual-detector SPECT/CT system (Symbia T6 or Symbia T16; Siemens K.K., Tokyo, Japan) while the patient breathed normally. Each detector was continuously and repeatedly rotated in the clockwise and counterclockwise directions across the same projection arc to obtain 360° projection data, and eight series of projection data (one per 32 s) were obtained during a 2-minute period. The acquired SPECT data (matrix size of 128×128, pixel size of 3.9 mm, slice thickness of 3.9 mm, energy window of 140 keV±10%) were then reconstructed using an iterative ordered-subsets expectation maximization algorithm (12 iterations, 6 subsets) and processed with Gaussian post-filtering (full width at half maximum, 3.9 mm). The matrix size, pixel size, and slice thickness in the reconstructed images were 128×128, 3.9 mm, and 3.9 mm, respectively. CT for attenuation correction and anatomical mapping with fusion images was also acquired using a low-dose protocol with 130 kVp and ref 50 mAs. CT images were reconstructed at a slice thickness of 3.0 mm and an image reconstruction interval of 1.5 mm. An image processing device (Syngo MI Applications VB10B; Siemens K.K.) was used for data collection and image reconstruction as reported previously.^30,31^

### Method of calculating ppo pulmonary function

#### SC method using Ali’s formula^12^

The SC method involved the following formula:

$${\text{\%ppoFEV}}_{1}={\text{preoperative \%FEV}}_{1}\times (1-\text{number of functional lung segments to be resected/total number of functional segments})$$

The total number of bilateral lungs is 19, of which 10 are in the right lung (3 in the upper, 2 in the middle, and 5 in the lower) and 9 are in the left lung (5 in the upper and 4 in the lower). The preoperative %FEV_1_ in the formula was substituted with the preoperative %DL_CO_ or %DL_CO_’ to calculate %ppoDL_CO_ and %ppoDL_CO_’.

#### Planar method

The fraction of total perfusion for the resected side was measured on the planar image of lung perfusion scintigraphy to correct differences in the perfusion fraction between the left and right lungs. The perfusion fraction value was then substituted into the following formula:^13,14^

$${\text{\%ppoFEV}}_{1}={\text{preoperative \%FEV}}_{1}\times (1-\text{fraction of total perfusion for resected-side lung}\times \text{number of functional lung segments to be resected/total number of functional segments for resected-side lung})$$

In this formula, (fraction of total perfusion for resected-side lung×number of functional lung segments to be resected/total number of functional segments for resected-side lung) indicates the predicted perfusion fraction of the lobe to be resected to the total perfusion of the preoperative whole lung. Additionally, (1–fraction of total perfusion for resected-side lung×number of functional lung segments to be resected/total number of functional segments for resected-side lung) represents the predicted perfusion fraction of the residual lung after lobectomy to the total perfusion of the preoperative whole lung.

The preoperative %FEV_1_ in the formula was substituted with the preoperative %DL_CO_ and %DL_CO_’ values to calculate %ppoDL_CO_ and %ppoDL_CO_’.

#### SPECT/CT

**Figure 1:**
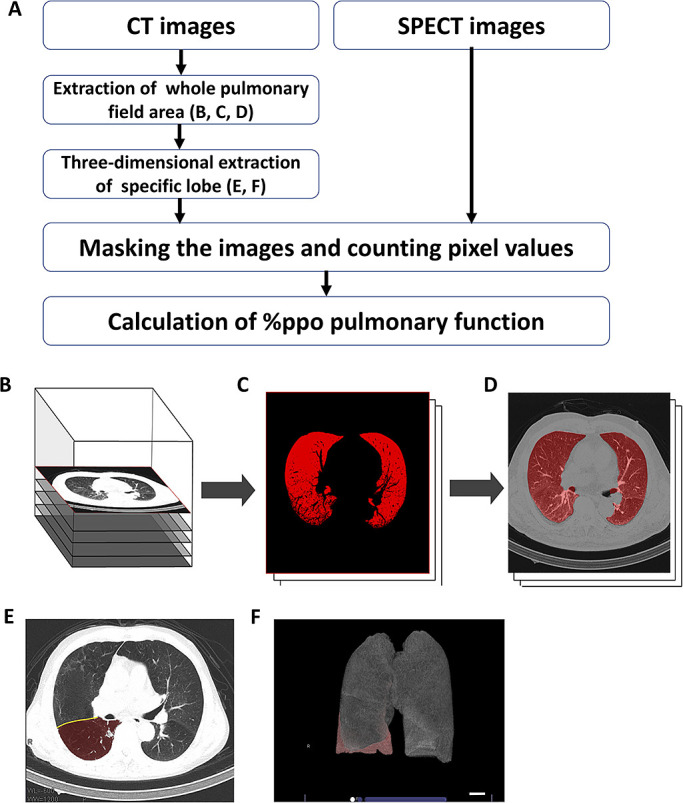
(**A**) Flowchart of single-photon emission computed tomography/computed tomography (SPECT/CT) method, (**B**–**D**) the process of extraction of the whole pulmonary field area, and (**E**, **F**) three-dimensional extraction of the specific lobe to be resected and the residual lung field area. %ppo, % predicted postoperative. (**B**) To extract the whole pulmonary field area from the original CT images, (**C**) each CT image underwent binarization processing with a fixed threshold, followed by (**D**) closing processing to repair defect parts with the vasculature and bronchi using Ziostation2 (Ziosoft Inc., Tokyo, Japan). (**E**) Interlobar lines were manually traced on several transaxial sections of the CT images that had undergone the binarization and closing processing. (**F**) Ziostation2 then automatically extracted three-dimensional images of the specific lobe to be resected and the other residual lung field area. The fraction of perfusion for the lobe to be resected was calculated by masking processing of the extracted three-dimensional CT data and the SPECT scintigraphy images followed by counting of the ratio of pixel values of the lobe to be resected and the whole lung field.

We calculated the fraction of perfusion for the lobe(s) to be resected using software that we developed ourselves from SPECT and CT lung perfusion scintigraphy images obtained simultaneously using a dual-detector SPECT/CT system (Symbia T6 or Symbia T16; Siemens K.K.). Figure 1 shows a flowchart that overviews the method we used. First, we used the Ziostation2 program (Ziosoft Inc., Tokyo, Japan) to extract the whole pulmonary field area from CT images via binarization processing (Figure 1C) and closing processing (Figure 1D). Next, we used Ziostation2 to manually trace the interlobar lines on multiple transaxial sections on the CT images (Figure 1E), and three-dimensional images of the lobe to be resected and the lung fields to be preserved were automatically extracted by Ziostation2 (Figure 1F). This process was based on the method by Ue et al.^32^ Using a program we developed independently, we then performed masking processing on the three-dimensional CT images, which were automatically extracted, and the SPECT images of lung perfusion scintigraphy. These were used to calculate the fraction of perfusion for the lobe(s) to be resected based on the ratio of pixel counts of the lobe(s) to be resected and the entire lung field (Figure 1A).

The following formula was then used:

$${\text{\%ppoFEV}}_{1}={\text{preoperative \%FEV}}_{1}\times (1-\text{fraction of perfusion for lobe to be resected})$$

The preoperative %FEV_1_ in the formula was substituted with the preoperative %DL_CO_ and %DL_CO_’ values to calculate %ppoDL_CO_ and %ppoDL_CO_’.

### Statistical analysis

To assess the interobserver agreement between the two perfusion fraction measurements (%) for each lobe calculated using the SPECT/CT method after two general thoracic surgeons (Measurers A and B) independently traced the interlobar lines, the correlation and reproducibility coefficient were determined using Pearson correlation and Bland–Altman analysis.^33^ The limits of agreement between the two measurers were calculated as the mean difference±1.96 multiplied by the SD between the two measurements. The absolute values of the differences between the measurements obtained by the two measurers were also calculated.

The predictive capability of the three prediction methods, in terms of correlation and the limits of agreement between the postoperative observed % values and %ppo values for FEV_1_, DL_CO_, and DL_CO_’, was assessed using Pearson correlation and Bland–Altman analysis. The absolute values of the differences between the postoperative observed % values and %ppo values obtained with the three methods were also compared.

Means of paired groups were compared by paired t-tests using Microsoft^®^ Excel for Mac version 16.24. For correlation data, Pearson’s r correlation coefficient was calculated using GraphPad Prism 5 for Mac OSX. Differences in the incidence of postoperative respiratory complications between two groups were compared by Fisher’s exact test using GraphPad Prism 5. Two-tailed analysis was considered significant at *p*<0.05.

## Results

**Figure 2:**
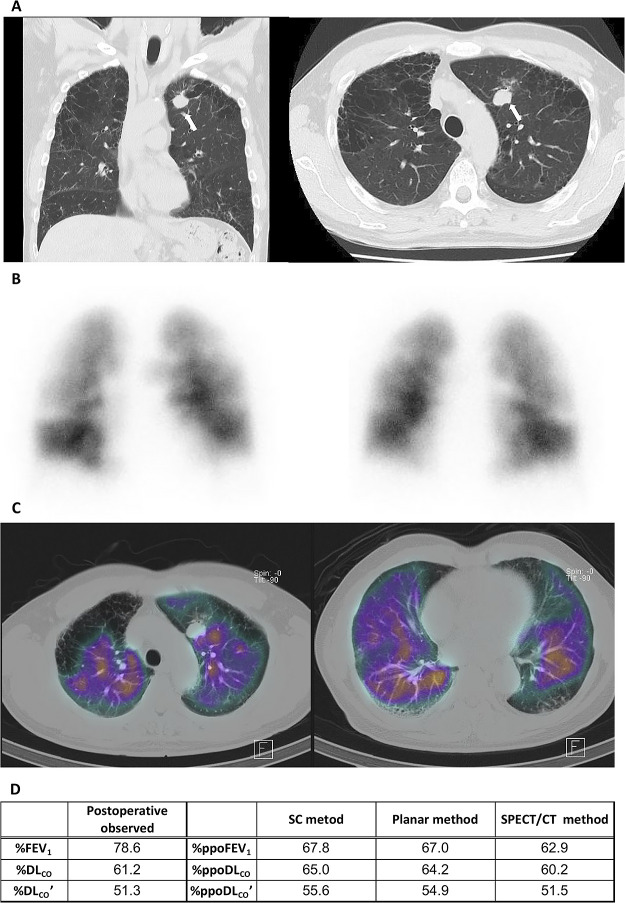
A 67-year-old man with invasive adenocarcinoma in the left upper lobe complicated by mild chronic obstructive pulmonary disease and idiopathic interstitial pneumonia. (**A**) Coronal (*left)* and axial (*right)* computed tomography (CT) images show a nodule with spiculations in the left upper lobe (*arrow)*, which was diagnosed as invasive adenocarcinoma. Note the heterogeneously distributed pulmonary emphysematous changes and reticulations. (**B**) Anterior (*left)* and posterior (*right)* planar images of lung perfusion scintigraphy demonstrate heterogeneously reduced uptake in the bilateral lung fields. (**C**) Perfusion single-photon emission CT (SPECT) images show extremely heterogeneous uptake of radioisotope in the bilateral lungs. (**D**) Postoperative observed % values and % predicted postoperative (ppo) values obtained with the simplified calculating (SC), planar, and SPECT/CT methods for the forced expiratory volume in 1 second (FEV_1_) and diffusing capacity for carbon monoxide (DL_CO_ and DL_CO_’).

A representative case is shown in Figure 2.

**Figure 3:**
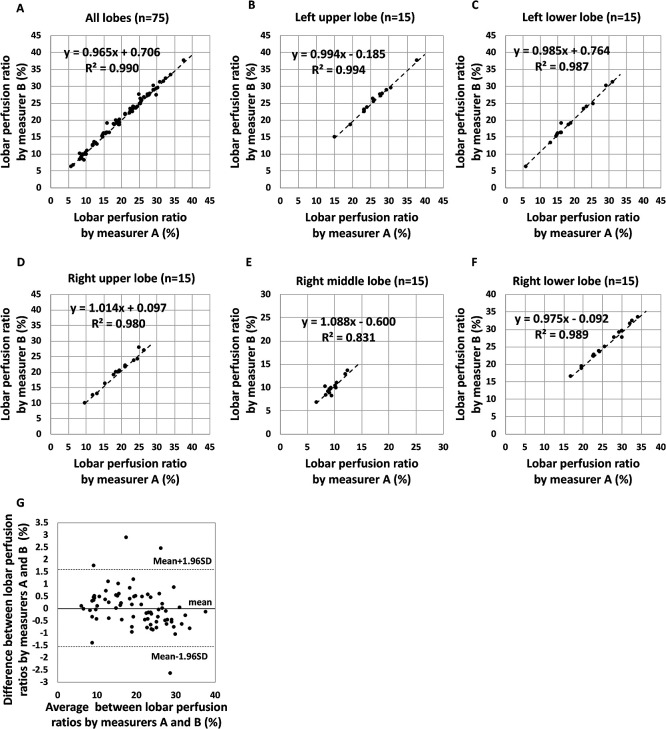
(**A**–**F**) Relationship between lobar perfusion ratio (%) calculated using the single-photon emission computed tomography/computed tomography (SPECT/CT) method with tracing of interlobar lines by Measurer A and that by Measurer B of (**A**) all lobes (n=75), (**B**) left upper lobe (LUL) (n=15), (**C**) left lower lobe (LLL) (n=15), (**D**) right upper lobe (RUL) (n=15), (**E**) right middle lobe (RML) (n=15), and (**F**) right lower lobe (RLL) (n=15). The dashed line represents the regression line. Note the significant correlation between values independently calculated by the two measurers at Pearson’s r of 0.995, 0.997, 0.993, 0.990, 0.912, and 0.994 for (**A**) all lobes, (**B**) LUL, (**C**) LLL, (**D**) RUL, (**E**) RML, and (**F**) RLL, respectively, and two-tailed *p* values of <0.0001 for either all lobes or each lobe. (**G**) Limits of agreement for the perfusion ratio within each lobe assessed by Measurers A and B using SPECT/CT. The mean difference (solid line) is 0.0%, and the upper (top dashed line) and lower (bottom dashed line) limits of agreement range from –1.6% to 1.6%. Dots denote data points.

Figure 3A to F shows a scatter diagram for a preliminary experiment in which the perfusion ratio (%) for each lobe of 15 patients was calculated using SPECT/CT after Measurers A and B independently traced the interlobar lines. Pearson’s r correlation between measurements obtained by the two measurers were as follows: all lobes=0.995 (*p*<0.0001), left upper lobe=0.997 (*p*<0.0001), left lower lobe=0.993 (*p*<0.0001), right upper lobe=0.990 (*p*<0.0001), right middle lobe=0.912 (*p*<0.0001), and right lower lobe=0.994 (*p*<0.0001). Although the right middle lobe tended to have a slightly lower Pearson’s r than the other lobes, the overall results were extremely good. The slopes of the regression lines were very close to 1, and all intercepts were <1. The limits of agreement (mean±1.96SD) for the perfusion ratio within each lobe assessed by Measurers A and B were 0.0%±1.6% (Figure 3G). The absolute values of the differences (mean±SD) between the measurements obtained by the two measurers were 0.57%±0.55% (maximum of 2.89% [right middle lobe], minimum of 0.00%). Based on the above analysis, we determined that the interobserver agreement of the data calculated using SPECT/CT was acceptable.

**Figure 4:**
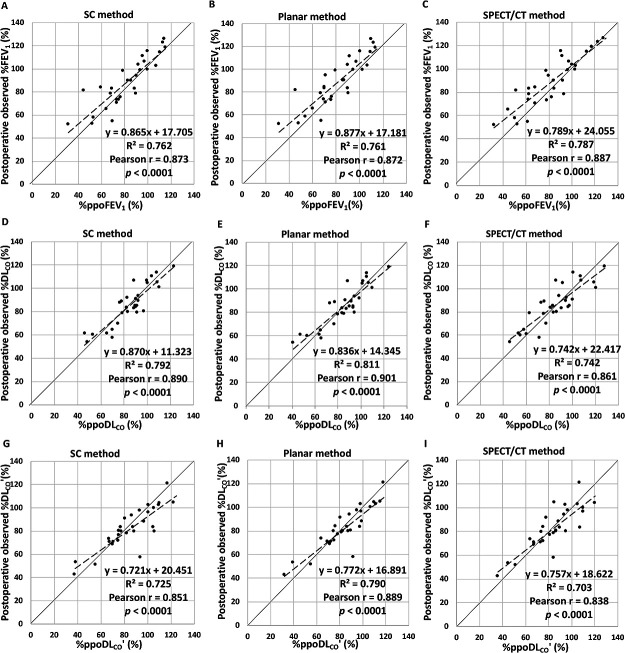
Relationship between postoperative observed % values for (**A**–**C**) forced expiratory volume in 1 second (FEV_1_) (n=30) or (**D**–**I**) diffusing capacity for carbon monoxide (DL_CO_, n=30 in **D**–**F**; DL_CO_’, n=29 in **G**–**I**) and % predicted postoperative (ppo) values for FEV_1_, DL_CO_, or DL_CO_’ calculated by the (**A**, **D**, **G**) simplified calculating (SC) method, (**B**, **E**, **H**) planar method, or (**C**, **F**, **I**) single-photon emission computed tomography/computed tomography (SPECT/CT) method. The solid line that crosses each figure diagonally indicates y=x. The dashed line represents the regression line. Determination coefficients (R^2^), Pearson’s r, and two-tailed *p* values are shown in each figure. (**A**–**C**) Regarding the prediction of postoperative FEV_1_, there seemed to be no differences in dispersion among the SC, planar, and SPECT/CT methods as evidenced by the respective R^2^ of 0.762, 0.761, and 0.787. (**D**–**F**) The SPECT/CT method seemed to have slightly more dispersion than the SC and planar methods for DL_CO_ (R^2^=0.742 vs. 0.811 and 0.792) and (**H**, **I**) slightly more dispersion than the planar method for DL_CO_’ (R^2^=0.703 vs. 0.790).

Next, we created a scatter diagram for the %ppo values of the pulmonary function test parameters (%ppoFEV_1_, %ppoDL_CO_, and %ppoDL_CO_’) obtained with the SC, planar, and SPECT/CT methods based on the preoperative pulmonary function test results (x axis) and the observed % values measured 2 to 4 months after surgery (postoperative observed %FEV_1_, %DL_CO_, and %DL_CO_’; y axis) (Figure 4). The correlation between the predicted %ppo values and postoperative observed % values for all three methods was satisfactory (Pearson’s r≥0.8, two-tailed *p*<0.0001). A more detailed observation of the scatter diagrams indicated that the variability for the postoperative FEV_1_ predictions did not differ among the three methods and that there were no clear differences in the coefficients of determination for the regression lines (R^2^) among the three methods (SC, 0.762; planar, 0.761; SPECT/CT, 0.787) (Figure 4A–C). For DL_CO_, however, the scatter diagram for the SPECT/CT method had slightly more variability than the SC and planar methods, and a larger %ppoDL_CO_ value appeared to be associated with a greater discrepancy (Figure 4D–F). The SPECT/CT method tended to have a smaller regression line R^2^ (0.742) than the other two methods (SC, 0.792; planar, 0.811). Observation of the postoperative DL_CO_’ prediction showed that the scatter diagram of the SPECT/CT method had slightly more variability than that of planar method but was nearly the same as that of the SC method (Figure 4G–I). The planar method tended to have a larger regression line R^2^ (0.790) than the other two methods (SC, 0.725; SPECT/CT, 0.703).

**Figure 5:**
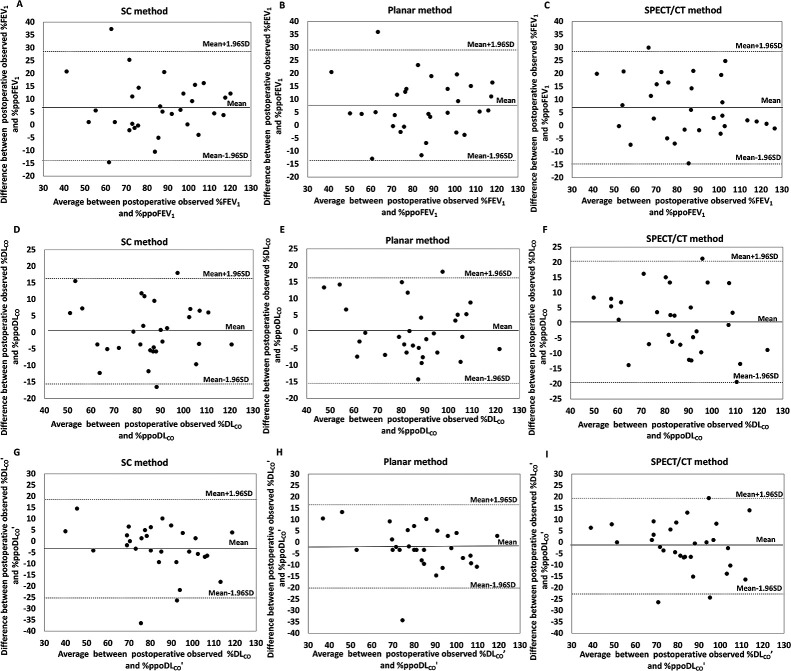
Graphs of Bland–Altman analysis of postoperative observed % values and % predicted postoperative (ppo) values for (**A**–**C**) forced expiratory volume in 1 second (FEV_1_) (n=30) and diffusing capacity for carbon monoxide (DL_CO_, n=30 in **D**–**F**; DL_CO_’, n=29 in **G**–**I**). The ppo values were calculated by the (**A**, **D**, **G**) simplified calculating (SC) method, (**B**, **E**, **H**) planar method, and (**C**, **F**, **I**) single-photon emission computed tomography/computed tomography (SPECT/CT) method. The solid horizontal line represents the mean difference. The top and bottom dashed lines indicate the upper and lower limits of agreement, respectively, and the dots denote data points.

The mean difference and limits of agreement of the three prediction methods between the postoperative observed % values and %ppo values for FEV_1_, DL_CO_, and DL_CO_’, which were determined using Bland–Altman analysis, are shown in Table 2 and Figure 5. The mean differences between the observed and %ppo values of FEV_1_ and DL_CO_ obtained with the three methods were comparable, whereas the mean difference (mean±SE) between the observed and %ppo values of DL_CO_’ obtained with SPECT/CT (–1.8±2.0) tended to be less than those obtained with the SC (–3.4±2.1) and planar (–2.4±1.8) methods. The limits of agreement between the postoperative observed % values and %ppo values for FEV_1_, DL_CO_, and DL_CO_’ did not differ among the three methods.

**Figure 6:**
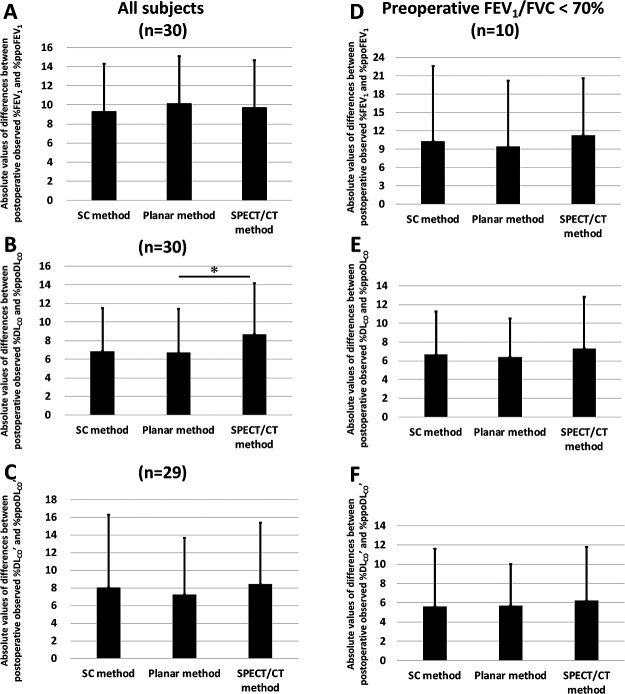
Absolute values of differences between postoperative observed % values and % predicted postoperative (ppo) values for (**A**, **D**) forced expiratory volume in 1 second (FEV_1_) and (**B**, **C**, **E**, **F**) diffusing capacity for carbon monoxide (DL_CO_ in **B** and **E**; DL_CO_’ in **C** and **F**) of all patients (**A**, **B**, n=30 each; **C**, n=29) and patients with obstructive ventilatory impairment (FEV_1_/FVC of<70%) (**D**, **E**, and **F**, n=10 each). The ppo values were calculated by the simplified calculating (SC), planar, and single-photon emission computed tomography/computed tomography (SPECT/CT) methods. Each column represents the mean value. The Y error bar represents the standard deviation (SD). (**A**) Absolute values of differences between postoperative observed %FEV_1_ and %ppoFEV_1_ for all patients were comparable among the SC, planar, and SPECT/CT methods. (**B**) Absolute values of differences between postoperative observed %DL_CO_ and %ppoDL_CO_ for all patients were not different between the SC and planar methods, whereas the values of the SPECT/CT method were significantly higher than those of the planar method (*p<0.05). (**C**) Absolute values of differences between postoperative observed %DL_CO_’ and %ppoDL_CO_’ for all patients were not significantly different among the three methods. There were no statistically significant differences between postoperative observed % values and %ppo values for (**D**) FEV_1_, (**E**) DL_CO_, and (**F**) DL_CO_’ in patients with obstructive ventilatory impairment among the methods.

Next, we compared the absolute values of differences between the postoperative observed % pulmonary function values and %ppo values among the three prediction methods (Figure 6A–C). The absolute values of the difference (mean±SD) in FEV_1_ did not significantly differ among the three methods (SC, 9.3±8.5; planar, 10.1±8.0; and SPECT/CT, 9.7±8.5) (Figure 6A). The absolute value of the difference in DL_CO_ obtained with the planar method (6.7±4.7) was significantly smaller than that obtained with the SPECT/CT method (8.7±5.5, *p*<0.05), and that obtained by the SC method (6.9±4.6) tended to be lower than that obtained with the SPECT/CT method (Figure 6B). No significant differences in the absolute values of the differences for DL_CO_’ were found among the three methods (SC, 8.0±8.3; planar, 7.2±6.4; and SPECT/CT, 8.4±7.0) (Figure 6C).

We then compared the absolute values of the differences between the postoperative observed % values and % ppo values in 10 patients with obstructive ventilatory impairment (FEV_1_%/FVC <70%) and found no significant differences among the three methods (Figure 6D–F). We conducted similar comparisons in patients with %FEV_1_ of <80% predicted but found no clear differences among the three methods in terms of FEV_1_ (n=4), DL_CO_ (n=4), or DL_CO_’ (n=3) (data not shown).

**Figure 7:**
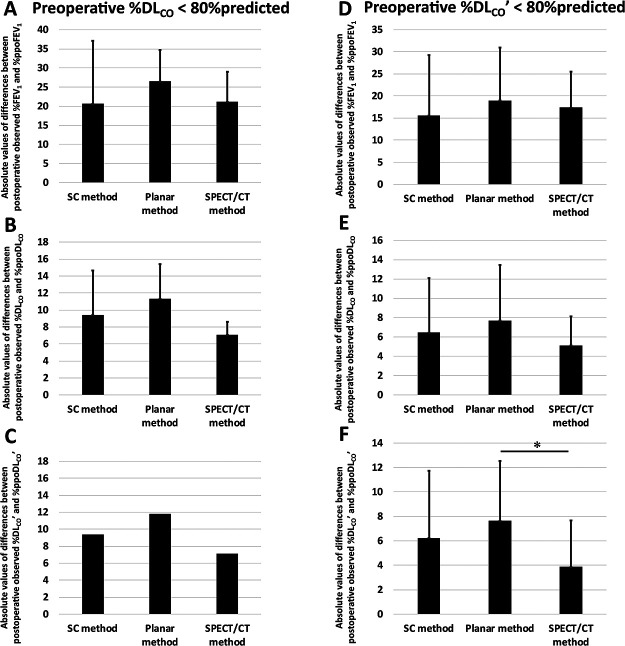
Absolute values of differences between postoperative observed % values and % predicted postoperative (ppo) values for (**A**, **D**) forced expiratory volume in 1 second (FEV_1_) and (**B**, **C**, **E**, **F**) diffusing capacity for carbon monoxide (DL_CO_ in **B** and **E**; DL_CO_’ in **C** and **F**) of patients with impaired diffusion capacity (**A**–**C**, preoperative %DL_CO_ of <80% predicted; **D**–**F**, preoperative %DL_CO_’ of <80% predicted). The ppo values were calculated by the simplified calculating (SC), planar, and single-photon emission computed tomography/computed tomography (SPECT/CT) methods. (**B**) Absolute values of differences between postoperative observed %DL_CO_ and %ppoDL_CO_ (n=3) and (**C**) those between postoperative observed %DL_CO_’ and %ppoDL_CO_’ (n=2) of the SPECT/CT method tended to be lower than those of the SC and planar methods in patients with preoperative %DL_CO_ of <80% predicted. (**E**) Absolute values of differences between postoperative observed %DL_CO_ and %ppoDL_CO_ of the SPECT/CT method tended to be lower than those of the SC and planar methods in patients with preoperative %DL_CO_’ of <80% predicted (n=4). (**F**) Absolute values of differences between postoperative observed %DL_CO_’ and %ppoDL_CO_’ of the SPECT/CT method were significantly lower than those of the planar method in patients with preoperative %DL_CO_’ of <80% predicted (n=4, *p<0.05).

We conducted similar testing in patients with pulmonary diffusion disturbance; i.e., %DL_CO_ of <80% predicted (Figure 7A–C) or %DL_CO_’ of <80% predicted (Figure 7D–F). There were no clear differences in FEV_1_ among the three methods (Figure 7A, n=3; D, n=5). In the patients with %DL_CO_ of <80% predicted, the absolute values of the differences between DL_CO_ (Figure 7B, n=3) and DL_CO_’ (Figure 7C, n=2) with the SPECT/CT method tended to be smaller than those with the other two methods (DL_CO_: SC, 9.4±5.2; planar, 11.3±4.1; and SPECT/CT, 7.1±1.5; DL_CO_’: SC, 9.4; planar, 11.8; and SPECT/CT, 7.1). In the patients with %DL_CO_’ of <80% predicted, the absolute value of the difference in DL_CO_ obtained with the SPECT/CT method tended to be smaller than those obtained with the other two methods (SC, 6.4±5.7; planar, 7.7±5.8; and SPECT/CT, 5.1±3.0; n=4) (Figure 7E). Notably, in the patients with %DL_CO_’ of <80% predicted, the absolute value of the difference in DL_CO_’ obtained with the SPECT/CT method (3.9±3.8) was significantly smaller than that obtained with the planar method (7.6±4.9, *p*<0.05) and tended to be smaller than that obtained with the SC method (6.2±5.5, n=4) (Figure 7F).

As described above, postoperative respiratory complications were observed in 5 of 30 patients (prolonged air leakage in 4 patients and chronic respiratory failure requiring home oxygen therapy in 1 patient) (Table 1). In accordance with the recommendations in the American College of Chest Physicians (ACCP) guidelines 2013,^1^ a patient is considered at high risk of lobectomy when the %ppoFEV_1_, %ppoDL_CO_, or %ppoDL_CO_’ is ≤60%. If the %ppoFEV_1_, %ppoDL_CO_, and %ppoDL_CO_’ are all >60%, the patient is considered at low risk of lobectomy. When using the SC method, the incidence of postoperative respiratory complications was 3 of 7 patients (42.9%) in the high-risk group and 2 of 23 patients (8.7%) in the low-risk group; when using the planar method, the incidence was 3 of 6 (50.0%) in the high-risk group and 2 of 24 (8.3%) in the low-risk group; and when using the SPECT/CT method, the incidence was 3 of 8 (37.5%) in the high-risk group and 2 of 22 (9.1%) in the low-risk group. Only the planar method showed a statistically significant difference between the two groups (Table 3). The sensitivity for postoperative respiratory complication predictions was the same for all three prediction methods, and the negative predictive value was also nearly the same; conversely, the planar method showed the best results for both specificity and positive predictive value (Table 3).

## Discussion

According to national totals compiled by the Japanese Association for Thoracic Surgery,^34^ there were 40,302 cases of lung resection for primary lung cancer in Japan during 2015 (a 1-year period), and 174 (0.43%) postoperative deaths occurred within 30 days postoperatively. The leading cause of death in these cases was interstitial pneumonia followed by pneumonia and respiratory failure. The top three causes of death were all respiratory complications. Therefore, careful preoperative prediction of postoperative respiratory complications is considered essential. The most important predictive factor for postoperative respiratory complications and mortality has traditionally been ppoFEV_1_.^1,12,13^ In recent years, ppoDL_CO_ has been regarded as having increased importance as a predictive factor for postoperative respiratory complications and mortality, completely independent from ppoFEV_1_.^1,3,35^ In general, measurements of lung diffusion capacity use two values: DL_CO_ and DL_CO_’. They differ in how to determine alveolar volume (VA) used in the equations for calculating these values. For the former, VA is calculated as the inspiratory vital capacity+residual volume, while for the latter, VA’ is calculated using the helium gas dilution rate measured at the same time as DL_CO_’ (single-breath method).^36^ Therefore, in patients with severe airway obstruction, VA’ measurements can be smaller than VA measurements and the calculated DL_CO_’ can be smaller than DL_CO_. When interpreting data, DL_CO_’ is usually considered the lower limit of the lung diffusion capacity. Thus, when considering lung resection, DL_CO_’ is conventionally used as a parameter in many cases. In the present study, both DL_CO_ and DL_CO_’ were analyzed.

Prediction of postoperative pulmonary function is initially performed using the SC method.^12,37^ Then, to correct differences in the perfusion fraction between the left and right lungs, the left/right lung perfusion ratio is generally measured using the planar image of lung perfusion scintigraphy and substituted in the equation.^13,14^ Recently, evidence has mounted that SPECT/CT images are superior to planar images in terms of the detection accuracy for various respiratory diseases such as acute pulmonary thromboembolism.^38,39^ Studies comparing the conventional prediction methods (SC and planar methods) and the SPECT/CT method in predicting lung function after major lung resection have been conducted.^18–24^ Moreover, recent studies have investigated the predictive capabilities for postoperative pulmonary function of other state-of-the-art radiological methods, including perfusion magnetic resonance imaging and quantitative CT,^25,26^ as well as an improved segmentation method using conventional planar scintigraphy images.^27^ However, the outcome measures used in these studies were mainly FEV_1_ and FVC. No previous studies used DL_CO_, which is an important predictive factor independent of FEV_1_.^10,11^ Thus, the present study is the first to include DL_CO_ as a predictive factor.

In the present study, we first calculated the perfusion fraction of the lobe to be resected using the existing dual-detector SPECT/CT system and the image analysis system Ziostation2. These systems were used to mask and measure the pixels in a specific area of scintigraphy images made using a manual process of extracting the specified lobe area by tracing the interlobar lines and using three-dimensional CT data obtained from a program we developed on our own. We examined the correlation between the lobar perfusion ratios (%) calculated by two measurers who independently performed the interlobar line tracing procedure manually, which is a process prone to interobserver discrepancies. The results showed good correlations, very low limits of agreement, and small absolute values of differences between the two measurements for all assessed lobes. Therefore, we determined that the interobserver agreement of this system was acceptable and proceeded to the actual analysis.

First, analysis of the correlation using the scatter diagrams between the %ppo values and postoperative observed % values in all patients showed good correlations for FEV_1_, DL_CO_, and DL_CO_’ regardless of which method was used (Pearson’s r≥0.8, two-tailed *p*<0.0001). Particularly in regard to FEV_1_, we found no differences in the variability among the three prediction methods, and the coefficients of determination (R^2^) for the regression lines were nearly identical. Additionally, the limits of agreement for %ppoFEV_1_ did not differ among the three methods. The mean differences for the observed and %ppo values of FEV_1_ obtained with the three methods were comparable, and all were positive values, indicating that %ppoFEV_1_ tended to be underestimated regardless of the prediction method used. We next compared the absolute values of the differences between the postoperative observed %FEV_1_ and %ppoFEV_1_ and found no differences among the three methods. Previous studies have shown that the SPECT/CT method predicted ppoFEV_1_ more accurately than the conventional methods,^22–24^ while other studies failed to show superiority of the SPECT/CT method.^18,19^ Likewise, we did not show superiority of the SPECT/CT method in the present study.

However, in terms of DL_CO_ prediction, the scatter diagram for the SPECT/CT method had a greater level of variability than the SC method or the planar method, and the variability tended to increase as the %ppoDL_CO_ value increased. Conversely, the limits of agreement for %ppoDL_CO_ were not significantly different among the three methods. As with the correlation analysis findings, the absolute value of the difference between the postoperative observed %DL_CO_ and %ppoDL_CO_ obtained with the SPECT/CT method tended to be greater than those obtained with the other two methods. Particularly, this difference was statistically significant between the SPECT/CT method and the planar method. Pulmonary perfusion is affected by gravity, and the blood flow distribution changes depending on body position.^40–42^ Because the radioisotope is injected with the patients in the supine position, blood flow is greater in the dorsal lungs.^41^ One possible reason for the observed difference is that uneven blood flow became non-negligible by three-dimensional analysis. However, the absolute value of the difference between the postoperative observed %DL_CO_ and %ppoDL_CO_ was nearly equal between patients who underwent resection of the right middle lobe (8.8%, n=5), which is located on the ventral side, and patients who underwent resections of other lobes (8.7%, n=25). Regardless, we were unable to demonstrate the superiority of the SPECT/CT method for DL_CO_ prediction.

Our investigation of DL_CO_’ indicated that, like FEV_1_, there were no differences in the scatter diagrams, limits of agreement, or absolute values of differences between the postoperative observed %DL_CO_’ and %ppoDL_CO_’ among the three prediction methods. All of the mean differences between the observed and %ppo values of DL_CO_’ were negative values, indicating that %ppoDL_CO_’ tended to be overestimated regardless of which method was used. Because SPECT/CT tended to produce mean differences closer to 0 than the other two methods, SPECT/CT avoided overestimation of postoperative DL_CO_’ compared with the conventional methods. In any case, we were unable to demonstrate obvious superiority of the SPECT/CT method.

Therefore, we decided to extract patients who presented with pulmonary function abnormalities for use in further investigation. First, we compared the absolute values of the difference between postoperative observed %values and %ppo values in the 10 patients with obstructive ventilatory impairment (FEV_1_%/FVC<70%) and the 4 patients with %FEV_1_<80% predicted, which indicates chronic obstructive pulmonary disease of stage ≥II (DL_CO_’ in 3 patients). The results showed no differences in FEV_1_, DL_CO_, or DL_CO_’ among the three prediction methods. Next, we performed similar comparisons in patients with pulmonary diffusion capacity impairment (%DL_CO_ or %DL_CO_’ of <80% predicted). Notably, the absolute values of the differences between the postoperative observed %DL_CO_’ and %ppoDL_CO_’ in the four patients with DL_CO_’ of <80% predicted were significantly smaller with the SPECT/CT method than with the planar method and tended to be smaller with the SPECT/CT than SC method. Similarly, in the comparison of the absolute values of differences in DL_CO_ in the patients with DL_CO_’ of <80% predicted, the values for DL_CO_ in the patients with DL_CO_ of <80% predicted and the values for DL_CO_’ in the patients with DL_CO_ of <80% predicted also tended to be smaller with SPECT/CT than with the other two methods. All four patients with preoperative %DL_CO_’ of <80% predicted had emphysema with irregular distributions. One of these patients had interstitial pneumonia with fibrotic changes predominantly in the subpleural area of the bilateral lower lobes. DL_CO_ is strongly affected by the pulmonary capillary bed. Therefore, especially for patients with emphysematous or interstitial changes by which the pulmonary capillary bed is destroyed unevenly, resulting in abnormal distribution of blood flow and diffusion impairment,^15–17,43,44^ SPECT/CT may be superior to the conventional methods in predicting the postoperative pulmonary diffusion capacity because it can directly measure the perfusion fraction of each lobe.

In the last analysis, we compared the accuracy of the three methods in prediction of the incidence of postoperative respiratory complications when patients with %ppoFEV_1_, %ppoDL_CO_, or %ppoDL_CO_’ of ≤60% were considered at high risk of lobectomy in accordance with the recommendations in the ACCP guidelines 2013.^1,5,45^ The results showed that the sensitivity and negative predictive value for all three methods were nearly identical. When predicting postoperative complications to determine whether surgery is indicated, it is important to identify all patients in whom complications may occur without missing any. In this sense, it is important to have high sensitivity, a low false-negative rate, and a high negative predictive value. The specificity and positive predictive value were highest in the planar method among the three methods.

Limitations of this study include its single-center retrospective design, small sample size, the relatively good pulmonary function in most patients, and measurement bias in the SPECT/CT method. The SPECT/CT method reportedly provides more accurate fusion images when images are made during cessation of respiration or when the images are synchronized with respiration.^21,46–48^ Because we obtained our SPECT/CT images during spontaneous breathing, the two images might not have been perfectly matched; this may have caused unclearness interlobar lines. Additionally, the pulmonary perfusion distribution is greatly affected by gravity.^40,41^ We injected the radioisotope and took the SPECT images with the patients in the supine position; thus, we did not consider the effects of gravity. This is a point to be improved in future studies.

The routine use of SPECT/CT in preoperative testing is not recommended because it would lead to increased costs and labor.^18,19^ Our analyses of all patients included in this study failed to demonstrate the superiority of SPECT/CT in predicting postoperative pulmonary function or respiratory complications. Rather, our findings suggest that the planar method is superior when used to predict DL_CO_ and respiratory complications. In this sense, we believe that routine performance of SPECT/CT on all patients is unnecessary. However, although the sample size was very small, our data suggest the possibility that SPECT/CT may be more accurate than the conventional methods in predicting the postoperative pulmonary diffusion capacity in patients with diffusion impairment, which is the most important risk factor for postoperative morbidity and mortality. Further studies are required to investigate the usefulness of SPECT/CT in patients with more specific inclusion criteria.

## Figures and Tables

**Table1:** Patient characteristics and postoperative complications

Age, years	66±9 (37–77)
Sex	
Male	20
Female	10
Histological subtype of lung cancer	
Adenocarcinoma	21
Squamous cell carcinoma	7
Adenosquamous carcinoma	1
Pleomorphic carcinoma	1
Surgery performed	
Left upper lobectomy	5
Left lower lobectomy	4
Right upper lobectomy	6
Right middle lobectomy	5
Right lower lobectomy	8
Right upper and middle lobectomy	1
Right middle and lower lobectomy	1
Comorbidities	
FEV_1_/FVC<70%	10
Interstitial pneumonia	1
Postoperative complications	
Prolonged air leakage^a^	4
Home oxygen therapy^b^	1

Data are presented as mean±standard deviation (range) or n. FEV_1_, forced expiratory volume in 1 second; FVC, forced vital capacity. ^a^Prolonged air leakage defined as Clavien–Dindo classification grade ≥IIIA (requiring re-drainage or pleurodesis). ^b^Chronic respiratory failure requiring home oxygen therapy.

**Table2:** Mean difference and limits of agreement of three prediction methods (SC, planar, and SPECT/CT methods) between postoperative observed % values and % predicted postoperative values for FEV_1_ and DL_CO_

	Mean difference (%) (mean±SE)	Limits of agreement (%) (mean±1.96SD)
%FEV_1_		
SC method	6.6±2.0	6.6±21.1
Planar method	7.2±2.0	7.2±21.0
SPECT/CT method	6.7±2.0	6.8±21.7
%DL_CO_		
SC method	0.2±1.5	0.2±16.2
Planar method	0.3±1.5	0.3±15.9
SPECT/CT method	0.4±1.9	0.4±20.0
%DL_CO_’		
SC method	–3.4±2.1	–3.4±21.7
Planar method	–2.4±1.8	–2.4±18.5
SPECT/CT method	–1.8±2.0	–1.8±21.3

%FEV_1_, % forced expiratory volume in 1 second; %DL_CO_ and %DL_CO_’, % diffusing capacity for carbon monoxide; SC method, simplified calculating method using Ali’s formula^12^; SPECT/CT, single-photon emission computed tomography/computed tomography; SE, standard error; SD, standard deviation.

**Table3:** Comparison of accuracy of three prediction methods (SC, planar, and SPECT/CT methods) for prediction of the incidence of postoperative respiratory complications

	SC method	Planar method	SPECT/CT method
Morbidity (postoperative respiratory complications)			
High-risk group	3/7 (42.9%)	3/6 (50.0%)	3/8 (37.5%)
Low-risk group	2/23 (8.7%)	2/24 (8.3%)	2/22 (9.1%)
Fisher’s exact test	NS	*p*<0.05	NS
Sensitivity	0.60	0.60	0.60
Specificity	0.84	0.88	0.80
Positive predictive value	0.43	0.50	0.38
Negative predictive value	0.91	0.92	0.91

SC, simplified calculating method using Ali’s formula^12^; SPECT/CT, single-photon emission computed tomography/computed tomography; high-risk group, %ppoFEV_1_≤60% or %ppoDL_CO_≤60% or %ppoDL_CO_’≤60%; low-risk group, %ppo FEV_1_>60% and %ppoDL_CO_>60% and %ppoDL_CO_’>60%; NS, no significance. (%ppoFEV_1_, % predicted postoperative forced expiratory volume in 1 second; DL_CO_ and DL_CO_’, diffusing capacity for carbon monoxide)
